# Proteomic analysis of brain and spinal cord tissue reveals distinct immune and mitochondrial processes between human and mouse ALS models

**DOI:** 10.1038/s41598-025-11466-0

**Published:** 2025-09-30

**Authors:** Alanna G. Spiteri, Joel R. Steele, Han-Chung Lee, Haijian Zhang, Jiaqi Sun, Ralf B. Schittenhelm, Chien-Hsiung Yu, Catriona McLean, Colin L. Masters, Benjamin Goudey, Liang Jin, Yijun Pan

**Affiliations:** 1 The Florey Institute of Neuroscience and Mental Health, Parkville, VIC 3052 Australia; 2 Florey Department of Neuroscience and Mental Health, The University of Melbourne, Parkville, VIC 3052 Australia; 3 Monash Proteomics and Metabolomics Platform, Department of Biochemistry and Molecular Biology, Monash Biomedicine Discovery Institute, Monash University, Clayton, VIC 3168 Australia; 4 The ARC Training Centre in Cognitive Computing for Medical Technologies, University of Melbourne, Carlton, VIC 3052 Australia; 5 School of Translational Medicine, Monash University, Melbourne, Australia; 6 Australia BioCommons, University of Melbourne, VIC 3052 Melbourne, Australia

**Keywords:** Motor neurone disease, Amyotrophic lateral sclerosis, Immune-mediated pathology, Mitochondrial dysfunction, Neurodegeneration, TMT-proteomics, Motor neuron disease, Immunology, Proteomics

## Abstract

Amyotrophic lateral sclerosis (ALS) is a fatal neurodegenerative disease resulting in the progressive loss of motor neurons in the brain and spine. More than 95% of cases are pathologically characterized by the cytoplasmic accumulation of hyperphosphorylated and ubiquitinated transactive response DNA-binding protein 43 (TDP-43). Multiple mouse models with TDP-43 accumulation have been developed, however, whether they recapitulate molecular features of ALS pathology is unclear. Given the lack of curative treatment for ALS, there is an urgent need to identify the precise biological processes contributing to disease pathogenesis for the development of effective therapeutic treatments. Thus, in this study we employed label-based untargeted proteomics to characterize the ALS proteome and related biological processes in the spinal cord and brain of TDP-43^Q331K^ mice, a transgenic mouse model of ALS and the motor cortex and the cervical, thoracic, and lumbar spinal cord regions from humans. In humans, we observed highly overlapping responses across the four tissues examined, primarily related to the upregulation of immune processes and the downregulation of mitochondrial function. In contrast, TDP-43^Q331K^ mice demonstrate a lack of enrichment for immune activation and the opposite regulation of mitochondrial processes. A meta-analysis of previously published mouse datasets identified the Ubqln2 knock-out mouse model as showing stronger parallels with our late-stage human ALS. Overall, this study provides in-depth analysis of the site-specific dysregulated proteomes and their associated functional processes across species. Thereby, identifying potential therapeutic targets while emphasizing the limitations of specific mouse models at certain timepoints in recapitulating ALS-related processes for future model development.

## Introduction

Amyotrophic lateral sclerosis (ALS) is a debilitating and fatal neurodegenerative disease, with an increasing incidence of 6 per 100,000 people worldwide^1–4^. The clinical presentation of ALS is highly heterogeneous, involving motor and/or cognitive dysfunction, with a diverse range of ages, sites of onset and rate of disease progression^5–9^. Mutations in genes such as superoxide dismutase 1 (SOD1), TAR DNA-binding protein (*TARDBP* coding for TDP-43 protein), ubiquilin2 (UBQLN2), fused in sarcoma (FUS), chromosome 9 open reading frame 72 (C9ORF72), cause 10% of familial cases of ALS, however, the majority of cases are sporadic (90%) without a family history of disease^10,11^.

Strikingly, 90% of sporadic ALS cases show TDP-43 pathology in the affected brain and spinal cord regions, irrespective of a genetic mutation in TDP-43^8,12^. TDP-43 is a nuclear DNA/RNA binding protein ubiquitously expressed in many tissues and conserved across species^8^. TDP-43 controls the expression and function of thousands of genes, including synaptic proteins, glutamate transporters, receptors and inflammatory proteins. Therefore, the loss or dysregulation of TDP-43 has wide-reaching effects^8^. In this context, multiple mouse models have been highly effective in establishing and interrogating the link between TDP-43 pathology and ALS. In this study, we examined a mouse model expressing human Q331K mutant TDP-43 (TDP-43^Q331K^), which has been described as a robust and reproducible model without confounding effects on gut motility^13^. These mice display enhanced cytoplasmic mutant TDP-43 expression from 8 weeks old, progressive motor dysfunction beginning at 3 months of age and a 43% reduction in hindlimb muscle mass by 6 months^12^.

The pathophysiology of ALS is highly complex and multifactorial^5^, presenting complex management challenges, as no curative treatment is available^11^. With neurological disorders considered the foremost public health challenge of our time having a major impact on health-care systems^9^, there is an urgent need to identify biomarkers and therapeutic targets for disease intervention. In this study, we used TMT label-based, untargeted proteomics to reveal key misregulated proteins in the spine and brain of TDP-43^Q331K^ mice, as well as in the motor cortex, cervical, thoracic and lumbar tissue from humans with ALS. Overall, this study identifies important site-specific functional processes that are dysregulated in ALS tissue, providing clues for therapeutic targets. It also highlights the biological processes that are not recapitulated in TDP-43^Q331K^ mice and provides insight into alternative mouse models at specific disease stages that show a higher concordance to sporadic ALS in humans, thereby guiding future model development and selection.

## Materials and methods

### Human postmortem tissues

The use of postmortem human tissue was approved by The University of Melbourne Human research ethics committee. Human brain and spinal cord tissue was obtained from the Victorian Brain Bank, in part funded by The Florey, Fight Parkinson’s, FightMND, Ian and Maria Cootes and One More Night for Tania’ (Parkville, Victoria, Australia). The motor cortex, cervical, thoracic and lumbar tissue were analyzed from 5 control and 10 sporadic ALS cases. Gender and postmortem interval (PMI) are shown in Table 1.

**Table 1:** Summary of subject diagnosis and demographics.

Subject	Age	Gender	PMI	Diagnosis	Age of onset (years)	Disease duration (months)	Site of onset
1	54	Male	55	ALS	47	84	Limbs- upper arms
2	77	Male	42.5	ALS	75	16	Bulbar
3	65	Male	74	ALS	61	46	Lumbar – left lower limbs
4	78	Male	19	ALS	76	18	Bulbar
5	62	Female	47	ALS	60	24	Bulbar
6	79	Male	655	ALS	76.5	29	Bulbar
7	80	Female	57.5	ALS	78	20	Bulbar
8	63	Female	72	ALS	60	39	Bulbar
9	70	Female	34.5	ALS	68	27	Bulbar
10	76	Female	49	ALS	75	9	Bulbar & lower limbs
11	52	Male	33	Control	N/A		
12	76	Male	50	Control			
13	64	Male	68	Control			
14	78	Male	28	Control			
15	60	Female	57.5	Control			

### Animals and tissue collection

All animal experiments conformed to the Australian National Health and Medical Research Council published Code of Practice and were approved by the Florey Institute of Neuroscience and Mental Health Animal Ethics Committee (23–011-FINMH). Female transgenic mice expressing human TDP-43^Q331K^ (B6N.Cg-Tg(Prnp-TARDBP*Q331K)103Dwc/J, high-expressing line 103, stock number 017933) were obtained from the Jackson Laboratory (Bar Harbor, ME) and were maintained on a C57BL/6 J genetic background. As previously recommended, we used female mice as they show less variability in the various measurements requiring smaller group sizes required for statistical power^13^. Moreover, we used 6-month-old mice (symptomatic) as 10-month-old mice (later disease) do not provide significant additional information (i.e., neuroscoring, body weight, CatWalk hindlimb base of support and gastrocnemius/soleus muscle weight) over a study length of 6 months. All mice were housed with ad libitum access to standard rodent chow and water. Mice were euthanised with 1.5% isoflurane and transcardial perfusion with ice-cold PBS. Anesthesia depth was confirmed by absence of pedal reflex prior to initiating perfusion. Tissue was immediately cryopreserved and stored at −80 °C until further use. A total of 4 TDP-43^Q331K^ and 5 wildtype littermate mice were used at 180 days old. Due to observable behavioral differences between groups, experimenters could not be blinded to group allocation. However, mice of each genotype were randomly selected from breeding colonies for inclusion in the study.

### Proteomics

Brain and spinal cord samples were lysed in SDS lysis buffer (5% w/v sodium dodecyl sulphate, 20% SDS, 100 mM HEPES, pH 8.5), heated at 95 °C for 10 min and then probe-sonicated before measuring protein concentrations using a BCA kit. The lysed samples were denatured and alkylated by adding tris(2-carboxyethyl) phosphine hydrochloride and 2-chloroacetamide to a final concentration of 10 mM and 40 mM, respectively, and the mixture was incubated at 55 °C for 15 min. Samples were acidified by adding 12% phosphoric acid at 1:10 until pH reached around 2. The proteins were trapped using S-Trap mini columns (Profiti, Farmingdale NY), and S-Trap binding buffer (100 mM triethylammonium bicarbonate) at a buffer to protein ratio of 7:1 and centrifuged at 1500 x*g* for 2 min to bind protein to the column. The S-Trap was then washed four times with binding buffer to wash the trapped protein. Sequencing grade trypsin was added at an enzyme to protein ratio of 1:25 and incubated overnight at 37 °C. Tryptic peptides were sequentially eluted from the columns using (i) 100 mM triethylammonium bicarbonate, (ii) 0.2% v/v formic acid and (iii) 50% v/v acetonitrile, 0.2% v/v formic acid. The sequentially eluted fractions were pooled, concentrated in a vacuum concentrator and reconstituted in 40 µL of 200 mM HEPES, pH 8.5. Using a Pierce Quantitative Colorimetric Peptide Assay Kit (Thermo Scientific, Waltham, MA), equal peptide amounts of each sample were labelled with the TMTpro 16plex reagent set (Lot no: XJ351218, Thermo Scientific) according to the manufacturer’s instructions, considering a labelling strategy to minimize channel leakage. Individual samples were then pooled and high-pH RP-HPLC (1260 Infinity II, Agilent) was used to fractionate each pool into 12 fractions, which were acquired individually by LC–MS/MS to maximize the number of peptide and protein identifications. Human samples were acquired over four batches, with each tissue type analyzed in a single batch (10 ALS and 5 control tissues), on an Orbitrap Fusion Tribrid Mass spectrometer and Dionex Ultimate 3000 RSLCnano (Thermo Fisher Scientific) (as per^15^). Mouse samples were acquired over two batches, with each tissue type analyzed in a single batch (4 TDP-43^Q331K^ and 5 WT mice). Mouse samples were acquired with the Vanquish Neo LC system and Orbitrap Eclipse Tribrid mass spectrometer (Thermo Scientific) as per^14^. The mass spectrometry proteomics data have been deposited to the ProteomeXchange Consortium via the PRIDE [1]partner repository with the dataset identifier PXD062542.

### Data analysis

Pre-processing: The raw proteomics data files were analyzed with Proteome Discoverer 3.1 (Thermo Scientific) to obtain quantitative ms3 reporter ion intensities. Briefly; in built TMT-MS3 processing and consensus workflows were employed, Sequest HT with INFERYS rescoring, MsAmanda 3.0 and Percolator were utilized for protein and reporter ion quantification. PDv2.5 and only request was used for the human data. Samples were filtered for contaminants, non-confident proteins and missing values prior to imputation, sample loading normalization, ComBAT, batch correction and variance stabilization normalization in *TMT-analyst* developed by the Monash Proteomics and Metabolomics Platform (https://analyst-suites.org/apps/tmt-analyst/)^16–18^.

Differential expression analysis: Differential expression analysis was performed on the pre-processed and normalized data in R using linear mixed modelling with limma^19^. For the human dataset, PMI, age, sex, and site of disease onset were adjusted for in the final model. In the combined analysis, where all human CNS regions were pooled, tissue-specific and individual-specific effects were also accounted for, as each subject contributed data from four distinct regions and was therefore represented four times. For mice, only tissue type (i.e., brain and spine) was adjusted for in the final model, as all animals were the same sex and age. A cutoff of 0.05 of the adjusted p-value (Benjamini-Hochberg) along with a fold change of 1.5 was applied to determine significantly regulated proteins in the pairwise comparison. A list of all proteins and the differential expression analysis for each contrast is shown in Supplementary File 1.

Enrichment analysis: Gene set enrichment analysis (GSEA), over-representation analysis (ORA) and plot generation was performed in DEP2^20^, using clusterProfiler^21^ and enrichplot^22^. Three annotation databases were considered: Gene Ontology (separated into Biological Processes, Molecular Functions and Cellular Components)^23,24^, Kyoto Encyclopedia of Genes and Genomes (KEGG)^25^ and Reactome Pathways^26,27^. A cutoff of the adjusted p-value of 0.05 was considered significant with a minimum and a maximum gene size of 15 and 500, respectively for GSEA. Only significant terms were examined in plots. Significant terms and intersections across species and tissue are shown in Supplementary File 2. ORA was performed on all significant DEPs or significant up- and downregulated proteins. For GSEA, a normalized expression score (NES) > 0 was considered upregulated and NES < 0 was considered downregulated. To evaluate immune response terms, GSEA description terms were filtered:“immune”,“adaptive”,“innate”,“complement”,“antigen”,“cytokine”,“interleukin”,“leukocyte”, “B cell",“myeloid”,“neutrophil”,“T cell”and“interferon”. For mitochondrial-associated terms, the GSEA description column was filtered for:"ATP”,“oxidative”,“oxido”,“mitochon”, “NADH”, “respira” and “electron transport”. All terms were then individually assessed to determine their relevance to each category (Supplementary File 3).

Meta-analysis: The ProteomeXchange Consortium^28^ was used to identify previously published proteomics datasets of interest. Only mouse datasets were considered. ALS mouse models were identified by searching for keys acronyms and words including, “TDP-43”, “SOD1”, “FUS”, “C9orf72”, “Ubqln2”, “ALS”, “ALS” and “FTD", individually. Datasets were included if differential expression analysis was performed on the mouse model of interest versus an appropriate wild-type control. Twenty samples were collected from four studies examining mouse models of TDP-43, SOD1, C9orf72, FUS and Ubqln2 pathology across various timepoints and ages (see Table 2 for study details)^29–32^. Preprocessed and pre-ranked proteins were acquired from each study to ensure the resultant analysis was consistent with the original publication and that they were appropriately analyzed according to specific instrument and dataset requirements. These pre-ranked proteins were used to perform GSEA on each individual samples, as well as, together using clusterCompare with clusterProfiler^21^ in R.

**Table 2:** Summary of studies used in the mouse proteomics meta-analysis.

Gil et al., 2024, PMID: 38,374,041	
Mouse model	TDP-43 regulatable NLS8 (doxycycline (dox)-dependent inhibition of human cytoplasmic TDP-43 transgene)
Tissue type	Cortex
Sex	Female
Age	From 10 weeks of age
Experimental groups	1. Pre-onset—1 week off dox 2. Onset – 2 weeks off dox 3. Early disease – 4 weeks off dox 4. Late disease – 6 weeks off dox 5. Recovery – 6 weeks off dox and 2 weeks on dox
Phenotype	- Motor deficits emerge at early disease (4 weeks) with a decrease in muscle innervation, cortical neuron degeneration and astrogliosis - At late disease (6 weeks) there is microglial dysfunction, spinal cord motor neuron degeneration and a dramatic motor deficit - Mice in recovery show clearance of cytoplasmic TDP-43, restoration of endogenous TDP-43 in neurons, muscle re-innervation from remaining neurons, and regain motor skills

Gomes et al., 2024, PMID: 38,849,340	
Mouse model	1. **TDP-43:** B6;129S6Gt(ROSA)26Sortm1-(TARDBP*M337V/Ypet)Tlbt/J mice 2. **SOD1:** B6SJL-Tg(SOD1*G93A)1Gur/J mice 3. **C9orf72:** (Poly)GA-NES/C9orf72(R26(CAG-Isl-175GA) − 29 × Nes-Cre mice 4. **FUS:** Tg (Prnp-FUS)WT3Cshw/J mice
Tissue type	Pre-frontal cortex
Sex	Female & male
Age	1. 26 weeks 2. 14 weeks 3. 4.5 weeks 4. 4 weeks
Experimental groups	Female & male: 1. TDP-43 2. SOD1 3. C9orf72 4. FUS
Phenotype	- Pre-symptomatic stages (TDP-43 & SOD1 mice) - Early symptomatic (C9orf72)

Matveeva et al., 2023, PMID: 37,779,364	
Mouse model	1. **FUS:** ΔFUS(1–359) mice 2. **TDP-43:** B6.Cg-Tg(Prnp-TARDBP*A315T)95Balo/J)
Tissue type	Cortex
Sex	1. Female & male 2. Male
Age	1. PND 49–51 2. PND 57–62
Experimental groups	1. FUS 2. TDP-43
Phenotype	Presymptomatic

Whiteley et al., 2021, PMID: 33,277,362	
Mouse model	1. *Ubqln2* KO mice (generated by insertion of *loxP* sites flanking the one exon of murine *Ubqln2)* 2. *Ubqln2* KI containing mutant murine P520T *Ubqln2* allele corresponding to the human disease-causing P506T mutation
Tissue type	Brain, lumbar spinal cord or hippocampus
Sex	Male
Age	1. Young (4–6 months) and old (12–16 months) 2. Young (4–6 months)
Experimental groups	1. *Ubqln2* KO brain – young mice 2. *Ubqln2* KO brain – old mice 3. *Ubqln2* KO hippocampus – young mice 4. *Ubqln2* KO spine – young mice 5. *Ubqln2* KI hippocampus – young mice 6. *Ubqln2* KI spine – young mice
Phenotype	Animals have an intermediate, age-dependent neuromotor disease (10–14 months old) more severe than that seen in *Ubqln2* knock-in mice

Spiteri et al. 2025 (Current study)	
Mouse model	B6N.Cg-Tg(Prnp-TARDBP*Q331K)- 103Dwc/J mice
Tissue type	Brain or spine
Sex	Female
Age	PND 180
Experimental groups	1. Brain from TDP-43 mice 2. Spine from TDP-43 mice 3. Brain & spine (computationally merged)
Phenotype	- Enhanced cytoplasmic mutant TDP-43 expression from 8 weeks old - Progressive motor dysfunction beginning at 3 months of age - A 43% reduction in hindlimb muscle mass by 6 months^12^

Plot and graph generation: Unsupervised and supervised clustering using principal component analysis **(**PCA) and Partial Least Squares Discriminant analysis (PLS-DA), respectively was applied using the mixOmics package^33^ with default settings. Volcano plots were generated using DEP2^20^, Upset plots with UpsetR^34^, heatmaps with pheatmaps^35^ and gene-concept network, enrichment map and biological theme comparison in clusterProfiler^21^ in R.

## Results

### Mitochondrial and muscle functionality are dysregulated in TDP-43 mice

**Fig. 1:**
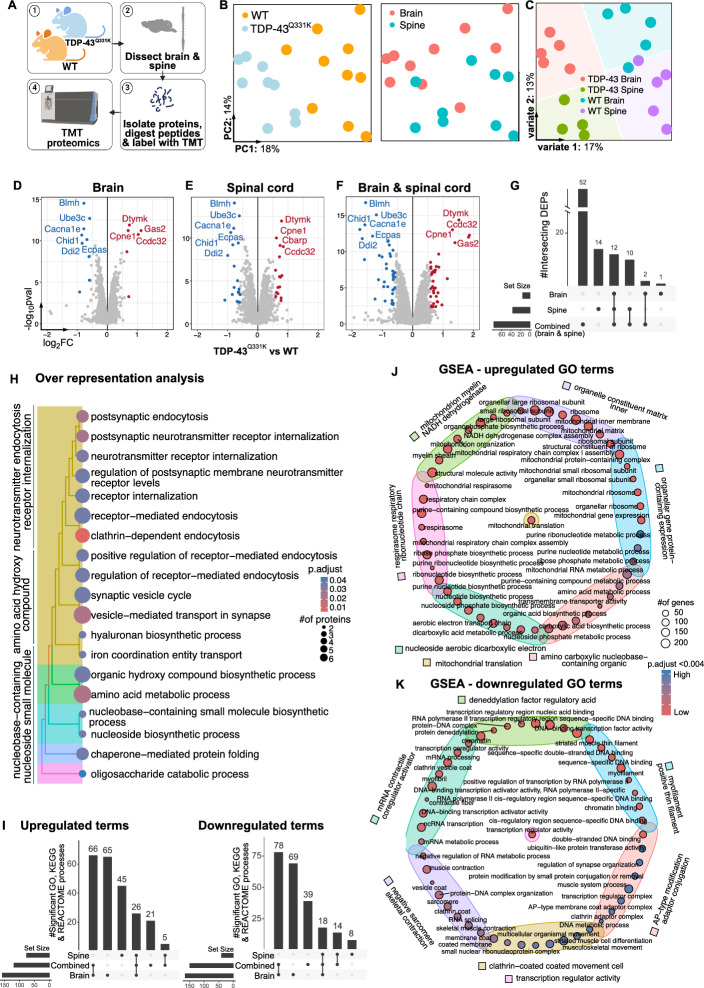
Mitochondrial and muscle functionality is dysregulated in TDP-43 mice. (**A**) Schematic of experimental design. The brain and spinal cord were dissected from wild type (WT) and TDP-43^Q331K^ mice. Proteins were then extracted for TMT proteomics. (**B**) Principal component analysis plot coloured by genotype and tissue type. (**C**) Partial Least Squares Discriminant analysis (PLS-DA) plot classed and coloured by genotype and tissue type. (**D**-**F**) Volcano plots showing up- and down-regulated proteins in TDP-43^Q331K^ mutant mice compared to WT controls in the brain (**D**), spinal cord (**E**) and brain and spine combined, representing a joint analysis accounting for tissue-specific variability (**F**). (**G**) Upset plot displaying the numbers of unique and overlapping significantly differentially expressed proteins (DEPs) across tissue types in TDP-43^Q331K^ mutant mice compared to WT controls. (**H**) Tree plot showing the significant gene ontology biological processes by overrepresentation analysis (ORA) with category labels. (**I**) Upset plots showing the number of significant up- and down-regulated gene set enrichment analysis (GSEA) processes across tissue type. (**J**, **K**) Enrichment map identifying the top 50 up- (**J**) and down- (**K**) regulated gene ontology processes and their grouped category labels.

To investigate the functional processes contributing to disease progression in ALS, we analyzed the brain and spinal cord tissue from WT and TDP-43^Q331K^ mice at postnatal day 180 using TMT label-based untargeted proteomic analysis (Fig. 1A). A total of 129,778 peptide groups (6.6 m PSMs) were identified and assigned to 11,572 protein groups (12,292 proteins), of which 7,946 were quantified across all samples.

Principal component analysis (PCA) and Partial Least Squares Discriminant analysis (PLS-DA) on all proteins demonstrated distinct separation of genotypes and tissue types, indicating differential proteomic responses (Fig. 1B, C). Using a linear model, we identified 36 and 15 significant differentially expressed proteins (DEPs) in the spine and brain, respectively (Fig. 1D & E** and **Supplementary File 1), with 93% of spine DEPs overlapping with brain DEPs (Fig. 1G). To compare all TDP-43^Q331K^ vs WT tissue, we combined the brain and spine data (i.e., “combined”) and adjusted for tissue-specific effects in our linear model. This identified a total of 76 DEPs in TDP-43^Q331K^ vs WT tissue (Fig. 1F & G).

To gain insight into the function of these deregulated proteins we performed Over Representation Analysis (ORA) (Fig. 1H). On the combined dataset (brain and spine) the 76 DEPs were associated with 128 significant Gene Ontology (GO), 4 KEGG and 20 REACTOME processes. The top GO processes were related to endocytosis, postsynaptic activity, neurotransmitter internalization and biosynthetic, metabolic and catabolic processes. These were grouped into five broad categories via pairwise similarities and hierarchical clustering of enriched terms, including 1) neurotransmitter endocytosis receptor internalization, 2) amino acid hydroxy compound, 3) nucleoside-containing nucleoside small molecule, 4) chaperone − mediated protein folding oligosaccharide catabolic process and 5) oligosaccharide catabolic process (Fig. 1H).

Next, using Gene Set Enrichment Analysis (GSEA) we examined the top up- and down-regulated processes in the entire protein set (Fig. 1I-K and Supplementary File 2). Interestingly, despite having less DEPs, the brain was associated with almost three-times more GSEA terms than the spine (332 vs 116 terms in the brain vs spine), with the majority of these being unique to the brain (134 terms) rather than shared (63 terms) with the spine. The unique brain processes were associated with respiration, mitochondrial function, oxidative phosphorylation, GABA B receptor activation, metabolism and ATP synthesis (Supplementary File 2). For the spine, these included nucleoside transport, pre- and post-synapse translation, extracellular matrix proteoglycans, complement cascade and neural cell adhesion molecule (NCAM) signaling for neurite outgrowth (Supplementary File 2). In the tissue-combined dataset, we observed an upregulation in DEPs relating to mitochondrial function, respiration and metabolic processes (Fig. 1J). In contrast, downregulated DEPs were associated with muscle differentiation, movement and contraction (Fig. 1K). Overall, while the spine showed the highest number of deregulated proteins, both the brain and spine demonstrate a shared alteration in mitochondrial and muscle functionality.

### Overlapping responses across human tissues affected by ALS

**Fig. 2:**
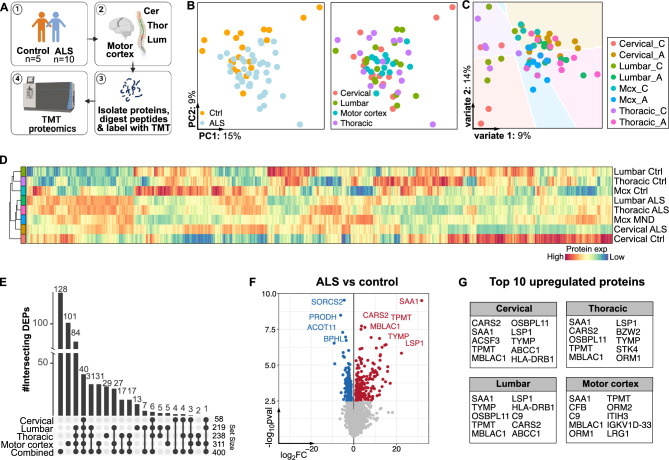
Proteomic signatures across ALS tissue are highly overlapping. (**A**) Schematic of experimental design. The motor cortex and cervical, lumbar and thoracic spinal cord tissue was obtained from 5 control and 10 ALS cases. Proteins were then extracted for TMT proteomics. (**B**) Principal component analysis plot coloured by condition and tissue type. (**C**) Partial Least Squares Discriminant analysis (PLS-DA) plot classed and coloured by condition and tissue type. (**D**-**F**) Heatmap showing the normalized, log2 and scaled protein expression values across tissue and condition. Hierarchical clustering was performed on rows (tissue) and columns (proteins). (**E**) Upset plot showing the number of unique and common significantly differentially expressed proteins (DEPs) in ALS versus control samples, for each tissue type. (**F**) Volcano plots showing up- and down-regulated proteins in all control versus all ALS tissue. (**G**) Top 10 upregulated proteins in each ALS tissue type compared to controls.

To examine whether our proteomic findings from TDP-43^Q331K^ mice could be generalized to humans, we obtained four different tissue regions from 10 ALS and 5 human control cases (Fig. 2A**)**. These included the motor cortex, cervical, thoracic and lumbar regions. Subjects ranged from 52–80 years of age with all ALS cases being sporadic and site of onset mainly occurring in the bulbar region (Table 1). The age of onset ranged from 47–76 years with the disease spanning 9–84 months.

A total of 102,196 peptide groups (464 k PSMs) were identified and assigned to 10,238 protein groups (11,277 proteins), of which 7,070 were quantified across all samples. By PCA, there was subtle segregation by disease status (i.e., ALS versus control), but none by tissue type (Fig. 2B). Using a supervised clustering method, PLS-DA, tissue regions only separate based on disease status when a background prediction is calculated (Fig. 2C). This implies that there could be consistent proteomic changes across tissue types in ALS. Supporting this, hierarchical clustering and heatmap visualization of all normalized proteins demonstrate that all ALS tissue cluster together and exhibit a similar pattern of protein expression (Fig. 2D). Control tissue, however, appeared to be more heterogeneous with cervical tissue clustering away from the other control regions (Fig. 2D).

Using a linear mixed model, we then performed differential expression analysis comparing control versus ALS cases on the combined and tissue-stratified data (Fig. 2E-G). In doing this, we adjusted for postmortem interval, sex, age, disease duration and site of pathology. Interestingly, the motor cortex showed the highest number of DEPs which were mostly unique to this tissue type (311 total DEPs and 101 unique DEPs) (Fig. 2E). Cervical, lumbar and thoracic tissue, however, had the least number of DEPs with 58, 219 and 238, respectively. These tissues had more shared than unique DEPs across all tissue types (87–100% shared DEPs) (Fig. 2E). We then combined all tissue regions and adjusted for tissue specific effects, identifying 400 DEPs in all ALS versus control samples. These include top upregulated proteins involved in immune regulation, response to cellular stress, inflammation (SAA1, CARS, MBLAC1, TYMP and LSP1) and drug metabolism (TPMT). The top downregulated proteins are associated with metabolic regulation of proline (PRODH), lipid (ACOT11) and energy metabolism (BPHL) and neurological function (SORCS2) (Fig. 2F & G and Supplementary File 1).

### Immune and mitochondrial functions are differentially regulated in ALS

**Fig. 3:**
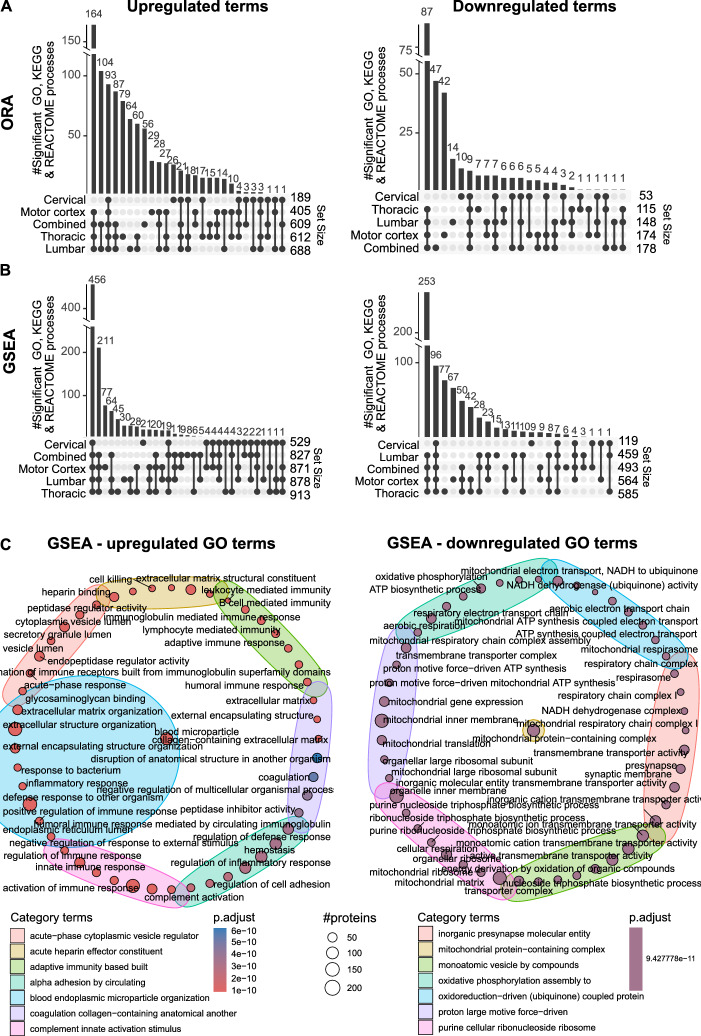
Immune and mitochondrial functions are differentially regulated in ALS. (**A**, **B**) Upset plots showing the number of significant up- and down-regulated terms across tissue type using overrepresentation analysis (ORA) (**A**) and gene set enrichment analysis (**B**). (**C**) Enrichment map identifying the top 50 up- and down-regulated gene ontology processes and their grouped category labels using GSEA.

We next performed ORA and GSEA on combined and tissue-stratified data to better understand the function of the DEPs and provide insight into mechanisms influencing disease progression (Fig. 3). Overall, more terms were upregulated than downregulated across all tissues and 85–97% of all GSEA terms were shared across tissue types, indicating common responses across regions (Fig. 3A & B). Strikingly, we observed 456 common upregulated GSEA processes across all tissues (Fig. 3B and Supplementary File 2). A list of all enriched processes in the combined and tissue stratified dataset is shown in Supplementary File 2.

Interestingly, of the top upregulated GO terms, the majority of these were associated with immune and inflammatory responses, including the innate and adaptive immune responses, B cell mediated immunity and complement activation (Fig. 3C and Supplementary File 2). Supporting this, the top KEGG and REACTOME terms related to the regulation of the complement and coagulation cascade (Supplementary File 2). Other notably top REACTOME terms include neutrophil degranulation, toll-like receptor cascades, antigen processing-cross presentation, cytokine signaling, adaptive immune response, interleukin-1 family and interferon gamma signaling (Supplementary File 2). Importantly, processes associated with the immune response were upregulated in all tissue regions, with the most terms identified in the thoracic region (175 terms, ~ 20% of all upregulated terms), followed by lumbar (162 terms), motor cortex (142 terms) and cervical tissue (113 terms) (Supplementary File 2).

In contrast to our findings in mice, the majority of the downregulated GO processes in the combined human dataset were involved in mitochondrial function, respiration, ATP synthesis and biosynthesis processes (Fig. 3C and Supplementary File 2). Correspondingly, the top downregulated KEGG and REACTOME processes were also associated with oxidative phosphorylation, complex I biogenesis, citric acid cycle and mitochondrial translation termination/elongation/initiation (Supplementary File 2). Examination of individual tissue types showed 82, 74, 72 and 37 mitochondrial-related terms downregulated in the lumbar, thoracic, motor cortex and cervical tissue, respectively (Supplementary File 2). Overall, across four human tissues affected by ALS, there were more shared enriched processes than unique and these related to immune and mitochondrial functions.

### TDP-43 mice do not recapitulate immune responses in ALS

**Fig. 4:**
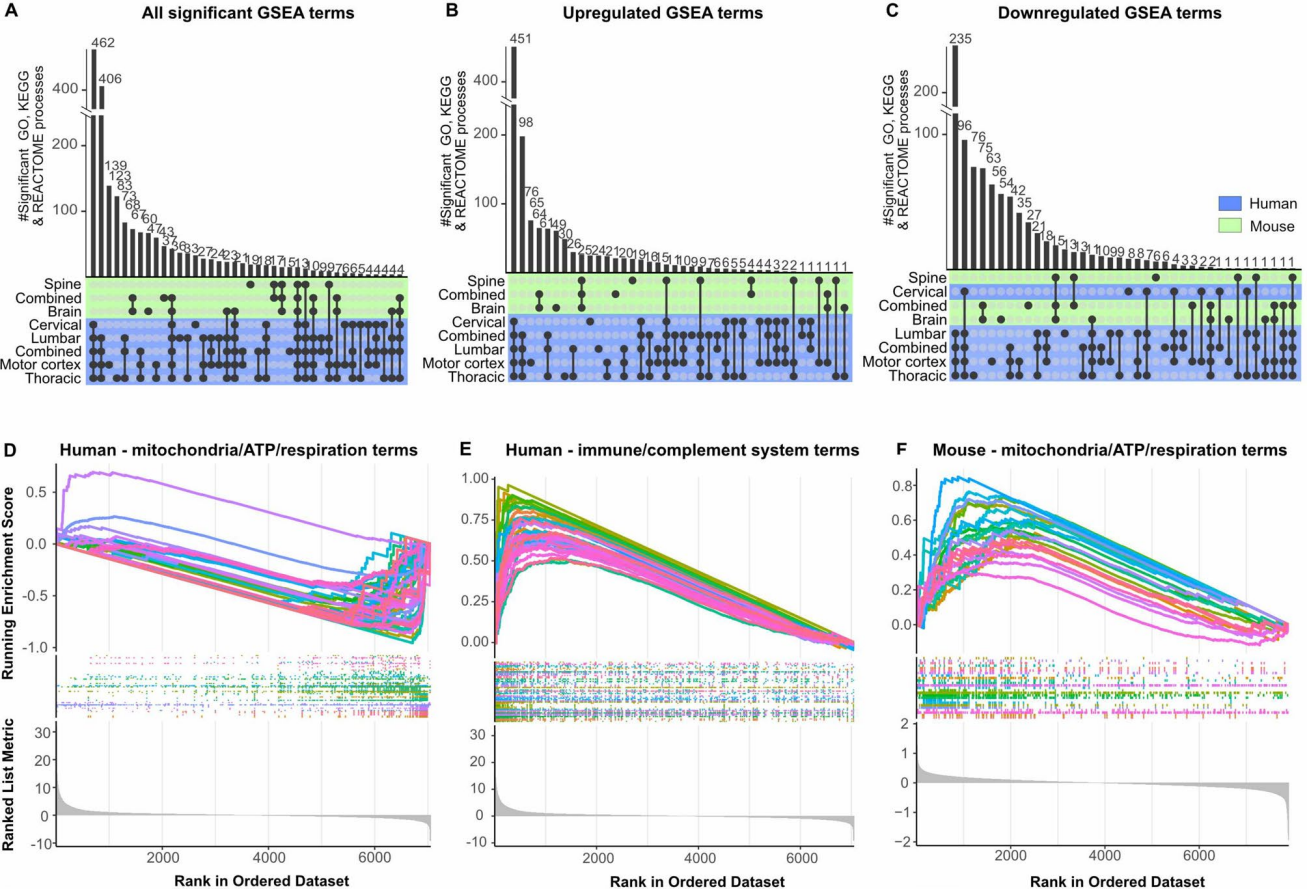
TDP-43 mice do not recapitulate inflammatory immune responses in ALS. (**A**-**C**) Upset plots showing the number of all significant (**A**), up- (**B**) or down-regulated (C) terms across tissue type and species using gene set enrichment analysis (GSEA). Set sizes are shown in the previous figures. (**D**-**F**) Rank plots showing the top terms associated with mitochondrial function (**D**, **F**) or the immune system (**E**) in the tissue-combined human (**D**, **E**) and mouse dataset (**F**).

To compare our findings from mice and humans we generated an upset plot evaluating the unique and shared GO, KEGG and REACTOME terms (Fig. 4). Not surprisingly most terms were species-specific. However, of the overlapping processes, the majority of these were shared between the mouse spine and the four human tissues (Fig. 4A-C).

Notably, the 15 processes shared across species were regulated in opposite directions. Specifically, these were all associated with mitochondrial function and were upregulated and downregulated in mice and humans, respectively (Fig. 4B & C and Supplementary File 2). Indeed, further inspection of the GO GSEA results identified a total of 75 terms (7%) associated with mitochondria and ATP production that were all downregulated in humans, except for “oxidoreductase activity, acting on peroxide as acceptor” (Fig. 4D and Supplementary File 3). In mice, we identified 33 corresponding terms (16%), which were all upregulated (Fig. 4F and Supplementary File 3). This discrepancy may be a species-specific phenomenon or potentially due to the alternate disease stages examined in mice relative to humans.

Another striking finding was the abundant upregulation of immune responses in humans which was not apparent in mice (Fig. 4E and Supplementary File 3). We identified a total of 152 upregulated immune-related GO processes (14%) in the combined human dataset and zero up- or downregulated terms in the combined mice dataset. While this may be reflective of ALS commodities in humans it nonetheless suggests that TDP-43^Q331K^ animals do not fully recapitulate ALS disease processes.

### Enrichment analysis of multiple mouse models identifies those that exhibit higher concordance with human ALS

**Fig. 5:**
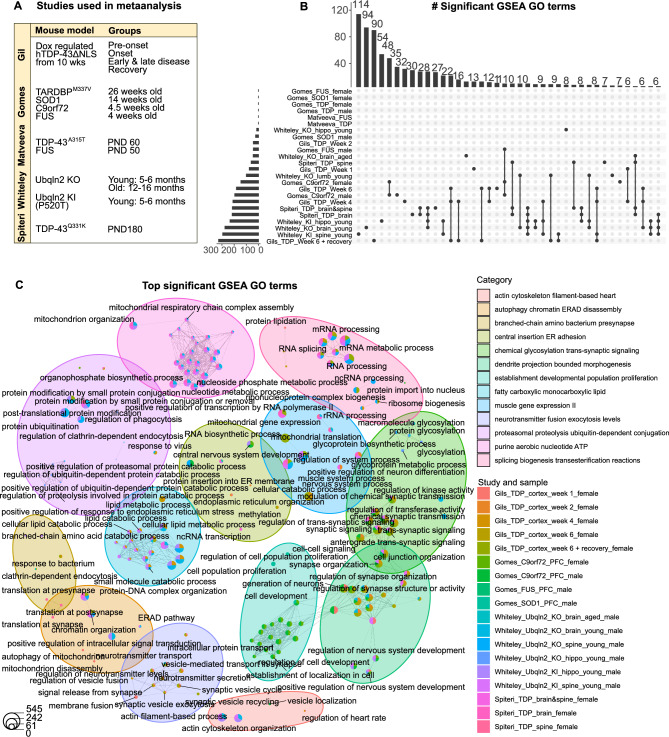
Enrichment analysis across mouse models of ALS. (**A**) Summary table of studies used in meta-analysis. (**B**) Upset plots showing the number of all significant GO terms (biological processes, cellular component and molecular function) based on GSEA on individual samples and studies. (**C**) Biological theme plot showing the top GO biological processes shared across each sample. The pie chart indicated the number of proteins in each term for each sample.

To identify mouse models of ALS that better recapitulate our findings in humans, we performed a meta-analysis using previously published proteomics datasets (Fig. 5). We filtered datasets on the ProteomeXchange Consortium^28^ for murine models of ALS and performed a GSEA on pre-ranked proteins from each study individually and together. Twenty samples were acquired from four studies profiling mouse models of TDP-43, SOD1, C9orf72, FUS and UBQLN2 pathology across various timepoints and ages (See Table 2 for a full list of study details) (Fig. 5A)^29–32^.

Across the 20 samples, 6 showed zero significantly enriched GO biological processes, cellular component or molecular function terms when performing a GSEA on each individual study (Fig. 5B). Of these included female FUS, SOD1 and TDP-43 mice from Gomes et al., as well as FUS and TDP-43 mice from Matveeva et al. Samples with significantly enriched terms mostly showed intersections between different timepoints, tissue types and sexes of the same mouse model (Fig. 5B). The largest intersection between studies was observed in 4 samples: the brain and spine combined from TDP-43^Q331K^ mice (from this study, i.e., Spiteri et al.) and hippocampus and brain from young *Ubqln2* KO mice (Whiteley et al.) (Fig. 5B). These terms were involved in mitochondrial and biosynthetic processes (Supplementary File 4).

We then performed a joint GSEA analysis on all samples together using the GO biological processes database. The most common upregulated terms across studies included those relating to mRNA processing and negative regulation of DNA-templated transcription, nucleobase-containing compound metabolic process, RNA metabolic and biosynthetic process (Fig. 5C** and **Supplementary File 5). While terms relating to synaptic and neurotransmitter function were the most common downregulated terms across studies (Fig. 5C** and **Supplementary File 5). Although it is important to note that this was not consistent across all samples and studies.

**Fig. 6:**
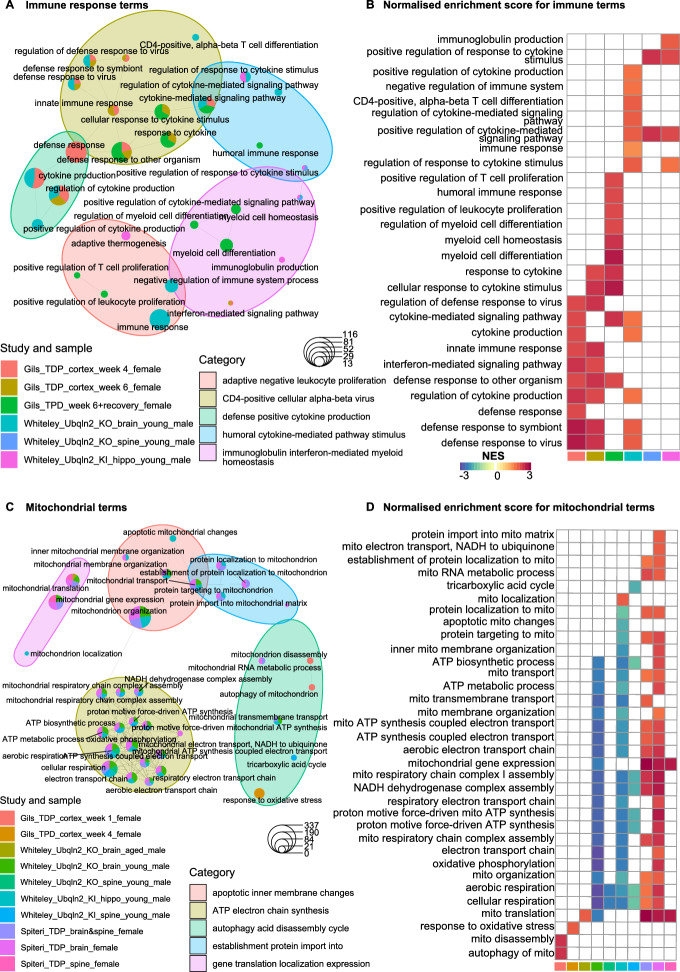
Alterations in mitochondrial and immune processes across mouse models of ALS. (**A**-**D**) Biological theme plot (**A**, **C**) and heatmap (**B**, **D**) showing the significant immune response (**A**, **B**) and mitochondrial-related (**C**, **D**) biological process terms enriched in mouse models of ALS.

Intriguingly, *Ubqln2* knock-out (KO) and knock-in (KI) models used in Whiteley et al., demonstrate parallels with top features identified in late-stage human ALS. Specifically, we identified an upregulation in immune processes and downregulation in mitochondrial processes (Fig. 6A-D). This was particularly true for the brain of young *Ubqln2* KO mice. Mitochondrial terms were also enriched in other studies (i.e., Gils et al., TDP-43 mice and TDP-43^Q331K^ from this study), however, these were inconsistently up- or down-regulated across samples (Fig. 6A-D). In contrast, of all the samples enriched for immune terms, these were all upregulated.

While we acknowledge that a direct side-by-side comparison of all mouse models using the same proteomics platform with appropriate batch controls is required to unambiguously identify unique and shared features across these model – our re-analysis of previously published data indicates that young *Ubqln2* KO mice recapitulate the top features identified in late-stage ALS in humans. However, further work is needed to characterize later timepoints across all mouse models, as discrepancies between human and mouse data may also stem from differences in disease stage.

## Discussion

ALS is a rapidly debilitating and fatal disease with no curative treatment available. Understanding the precise molecular processes contributing to disease pathogenesis is essential to reveal novel therapeutic targets. In this study, we identify the in-depth tissue- and species-specific proteomes to reveal dysfunctional biological processes linked to ALS.

Examination of the brain and spine of TDP-43^Q331K^ mice revealed overlapping and unique proteomic responses with deregulated proteins covering a range of cellular processes. These diverse pathways, including metabolism, signal transduction, gene expression, DNA repair, protein synthesis, cellular stress response, cell communication, adhesion, structure and transport, likely relate to the wide-reaching role of TDP-43 in regulating thousands of genes^8,9,36^. Interestingly, the downregulated proteins and associated GSEA terms were linked to muscle processes, synaptic function and neurotransmitter signaling. Downregulation in muscle functionality is potentially related to alterations in the neuromuscular junction and skeletal muscle denervation in ALS which causes muscle weakness and atrophy^37,38^. Additionally, as TDP-43 regulates synaptic and neurotransmitter proteins including synapsin I, synaptotagmin, glutamate transporters and receptors^39,40^, the downmodulation of synaptic function is likely associated with mutant TDP-43 expression in these mice. Correspondingly, it has been shown that neuronal stimulation increases the localization of TDP-43 to dendritic spines, indicating a role for TDP-43 in postsynaptic neurons^41^. Neurotransmitter imbalance has also been linked to astrocyte dysfunction impairing neuronal support and uptake of glutamate^40^. Importantly, this decrease in synaptic proteins has been verified in human ALS and FTD tissue and other mouse models of ALS using proteomics^31,42^.

While our analysis in mice provides an in-depth investigation of the precise processes dysregulated with mutant TDP-43 expression, ALS is far more complex and multifactorial than TDP-43 dysregulation. Our analysis of human ALS tissue spanning the motor cortex, cervical, thoracic and lumbar regions demonstrated many shared DEPs and GSEA terms with the top upregulated terms associated with the immune system. The role of the immune system in promoting inflammation and exacerbating neurodegeneration in ALS and many other neurological diseases, is now widely appreciated, particularly since many proteins encoded by mutant genes alter immune function^1,5,43^. Indeed, changes in circulating T cells and B cells responses, indicative of microglia activation in the brain, are one of the main sources of informative biomarkers for ALS detection and progression^44–46^. Further, the differential immune responses observed across patients are also thought to contribute to the expansive heterogeneity of the disease^1^. This could partially be due to different mutations or forms of disease inducing varying degrees of inflammation. Correspondingly, weighted co-expression network analysis demonstrated that C9orf72 expansion positive ALS patients had significantly more proteins associated with neuroinflammation compared to sporadic ALS cases^36^. Similarly, patients carrying SOD1 mutations had higher microglial activation than C9orf72 mutation carriers or sporadic ALS patients^47^. These previous studies and our results support the role of the immune system in amplifying pathology in patients with both familial and sporadic ALS^5^.

Of note, SAA1 was the top upregulated protein across all human ALS tissue. SAA1 is a member of the serum amyloid A family of apolipoproteins expressed during the initial response to infection and trauma. Consequently, it has been identified as biomarker of many infectious and inflammatory diseases including COVID-19, ischemic stroke, cancer and inflammatory bowel disease^48–56^. SAA1 is also upregulated in the brain of individuals with AD and multiple sclerosis^57,58^. Whether SAA1 originates from the periphery or the CNS in ALS remains unclear. However, it has been shown to prime microglia for IL-1β release^59^, potentially exacerbating inflammation in the ALS brain. Beyond microglial reactivity, this neuroinflammatory response seen in ALS has also been attributed to an alteration in peripheral immune cells, immune cell infiltration and systemic inflammation^5,42^. Efforts to suppress inflammation with immunosuppressive or immunomodulatory drugs alone in humans, however, have not altered the progression of disease^1^. The increased appreciation of immune cell specificity, along with their context- and temporal-specific functions, has contributed to the development of new therapeutic approaches in humans including the dual infusion of Tregs and IL-2 to harness their immunosuppressive ability^60^. While we have come a long way from using broader anti-inflammatory approaches, future investigations are required to unravel the precise cellular phenotypes, functions and interactions at specific timepoints to develop precise medication. This also necessitates stratified case monitoring, with immunological biomarkers for the precise tracking of immune responses in each patient^43^.

Importantly and in contrast to humans, our analysis revealed the limited inflammatory or immune related pathways upregulated in TDP-43^Q331K^ mice. We did, however, observe an upregulation in processes related to the complement system in the spine, as previously shown^61^. The limited upregulation of immune responses may be related to the timepoint we analyzed. However, this could also potentially be attributed to the fact the microglia are not highly expressing TDP-43^13^—which may be required to induce an inflammatory response. A previous study demonstrated that microglia were not overtly activated when TDP-43 was specifically induced in neurons and not microglia^47^. Additionally, glial cells expressing mutant transgenes in ALS models are sufficient to trigger motor neuron death and promote disease progression^43,62^. Nonetheless, considering Gil et al., demonstrated the upregulation of immune-related processes in their inducible TDP-43 mouse model^32^, the limited immune activation in our TDP-43^Q331K^ mice is highly likely a consequence of the model of TDP-43 and timepoint used. Analysis of a range of ALS mouse models at various timepoints is required to determine the contribution of the immune system in different transgenic mice to better model ALS pathology.

In mice and humans, mitochondrial processes were highly deregulated in ALS, albeit in the opposite direction. Mitochondrial function is crucial in the brain, consuming 20% of the body’s resting ATP production and providing an essential Ca^2+^ buffering organelle in neurotransmission^9^. The long lifespan of neurons and the essential role of mitochondria in the brain may make neurons more susceptible to damage and subsequent neurodegeneration following mitochondrial dysfunction. Importantly also, many proteins linked to ALS including SOD1, TDP-43, FUS, and C9ORF72, are known to interact with mitochondria leading to defects in function, potentially triggering disease onset and progression^9,63^. Indeed, suppression of TDP-43 localization to the mitochondria improved motor function in ALS transgenic mice^64,65^. Thus, mitochondrial dysfunction is a key driver of ALS considering its vital role in the brain to neuronal homeostasis.

The discrepancy in the differential regulation of mitochondrial processes in TDP-43^Q331K^ mice and humans could be due to the timepoint we examined in animals, necessitating investigations in older animals. Thus, this could suggest that early in disease, there is hyperactivity of cellular respiratory processes, which are later downmodulated as the disease progresses. Indeed, a previous study demonstrated differential alterations in mitochondrial dysfunction over the course of disease, with respiratory chain complex I proteins increased in pre-onset rNLS8 mice and decreased late in disease^32^. Growing evidence supports the notion that biological drivers of initiation and progression changes over the course of disease – with early mechanisms not necessarily reflected in the late stage. Similarly, proteomic examination of the human prefrontal cortex—an area exhibiting intermediate TDP-43 pathology at the time of death and used to provide insights into early disease mechanisms– demonstrated the opposite findings to the human dataset in this study^31^. Specifically, in humans they show that immune system activation was one of the top processes suppressed in females and males with ALS and that oxidative phosphorylation as one of the top activated processes. Thus, our findings of late-stage ALS and Gomes et al., early-stage disease, suggest that mitochondrial dysfunctions increase then decrease over the course of disease, while immune processes are activated late in disease. This is further reflected by Gils et., comprehensive time course investigation of rNLS8 mice^32^. Together, suggesting that TDP-43 accumulation and resulting mitochondrial hyperactivity may stimulate resident CNS cells later promoting inflammation, immune activation and dysregulation of metabolic processes.

Our analysis of previously published mouse proteomics datasets demonstrate that young *Ubqln2* KO mice show higher concordance with late-stage ALS. Specifically, these mice show a downregulation of mitochondrial function and upregulation of immune processes. UBQLN2 is a ubiquitin receptor thought to deliver ubiquitinated proteins to proteasomes for degradation^29^. Mutations in this protein result in familial FTD/ALS in humans through an unknown mechanism. It is important to note, however, that mouse models only represent specific features of disease. Thus, a major limitation of this study, and of the field more broadly, is the inadequate modelling of sporadic ALS^66^. This is primarily due to the reliance on transgenic mouse models carrying mutations associated with familial forms of the disease^67^. Future studies may need to identify proteins that accumulate in human sporadic ALS brains to better inform the selection or development of appropriate mouse models. This represents another key limitation of the current study as human tissue was not histologically profiled for specific protein aggregates. Emerging approaches, such as tissue organoids, humanized mouse models or induced pluripotent stem cells, may offer more accurate platforms for modelling sporadic ALS in the future. Nonetheless, transgenic mouse models have significantly enhanced our understanding of ALS. For instance, the C9orf72 model shows activation of immune and inflammatory pathways while the SOD1 model is enriched for the ERK1/2 cascade and response to oxidative stress and the TDP-43 model demonstrates alterations in transcription and endopeptidase activity^31^. Overall, this analysis has informed a noteworthy connection which warrants further investigation into the relationship between sporadic ALS/FTD and UBQLN2.

In this study, we demonstrated the broad proteomic dysregulation in bulk tissue, however future studies require investigations at a single-cell level to more precisely identify cell-specific responses. Moreover, our investigation is limited by the very small range of mouse models, sexes and timepoints examined, which prevented analysis of sex- and temporal-specific alterations in biological processes across various transgenic mice. Sex-specific alterations is an important differentiating factor in ALS and should be considered in future studies^31^. Finally, as ALS is a relatively rare and heterogeneous disease this has significantly challenged the acquisition of sufficient samples for analysis. Thus, validation with larger cohorts is essential to better understand ALS pathology for the development of effective treatments, taking into account differences between ALS originating in different sites and at various disease stages.

## Conclusions

Overall, we define the tissue-specific alterations in humans and mice with ALS associated with the modulation of diverse cellular processes. We suggest that mitochondrial dysfunction followed by immune activation are key drivers of pathology. The sheer complexity and heterogeneity of neurodegenerative diseases presents extreme challenges in targeting these pathologies. Future studies are required to more precisely define and track cell-specific immune dysregulation in humans for effective treatment.

## Supplementary Information

Supplementary Information 1.

Supplementary Information 2.

Supplementary Information 3.

Supplementary Information 4.

Supplementary Information 5.

## Data Availability

All data analysed during this study are included in supplementary information files. The mass spectrometry proteomics data have been deposited to the ProteomeXchange Consortium via the PRIDE [1] partner repository with the dataset identifier PXD062542.

## Abbreviations

ALS: Amyotrophic lateral sclerosis
C9ORF72: Chromosome 9 open reading frame 72
DEPs: Differentially expressed proteins
FUS: Fused in sarcoma
GO: Gene ontology processes
GSEA: Gene set enrichment analysis
ALS: Amyotrophic lateral sclerosis
ORA: Overrepresentation analysis
PCA: Principal component analysis
PLS-DA: Partial least squares discriminant analysis
SOD1: Superoxide dismutase 1
TARDBP: TAR DNA binding protein
WT: Wild-type
